# Survival After Cardiac Laceration From a Gunshot Wound: A Rare Case Report

**DOI:** 10.7759/cureus.50218

**Published:** 2023-12-09

**Authors:** Yusuke Tsukioka, Atsushi Nemoto, Raquel Salazar

**Affiliations:** 1 Cardiothoracic Surgery, University of Chicago Medicine, Chicago, USA; 2 Cardiac Support, University of Chicago Medicine, Chicago, USA

**Keywords:** acute pericardial effusion, bullet retention in pericardium, cardiac gunshot wound, cardiac laceration, hemodynamics response

## Abstract

In the United States, approximately 48,000 deaths annually are attributed to gunshot wounds, with a notably low mortality rate of 24.5% in cases involving cardiac injury. This case report presents a unique instance of a gunshot wound to the heart, where the patient, despite sustaining cardiac damage, maintained stable hemodynamics and underwent successful surgical removal of the bullet from the pericardial cavity. The absence of significant pericardial effusion and the maintenance of stable hemodynamics in this case provide valuable insights into the management of similar traumatic injuries. This report contributes to the existing knowledge on gunshot wound treatment, highlighting the importance of considering bullet retention in the pericardial cavity, even in the absence of substantial pericardial effusion.

## Introduction

In the United States, approximately 48,000 deaths occur annually due to gunshot wounds [1]. The mortality rate for gunshot wounds to the heart is reported to be low, at 24.5% [2]. This mortality rate is attributed to acute bleeding leading to cardiac tamponade or acute heart failure due to myocardial and valvular damage. We encountered a case where, despite a gunshot wound to the heart, the patient was transported to the operating room with stable hemodynamics, and a bullet was successfully extracted from the pericardial cavity. This case is presented with vivid preoperative and intraoperative images.

## Case presentation

The patient presented to the emergency department with a gunshot wound to the chest through the right upper arm. Computed tomography angiography (CTA) revealed trace hemopneumopericardium, a ballistic fragment within the pericardium, and hemopneumothorax. Emergency chest exploration was advised by cardiac surgery.

Preoperative examination

Physical Examination

**Figure 1:**
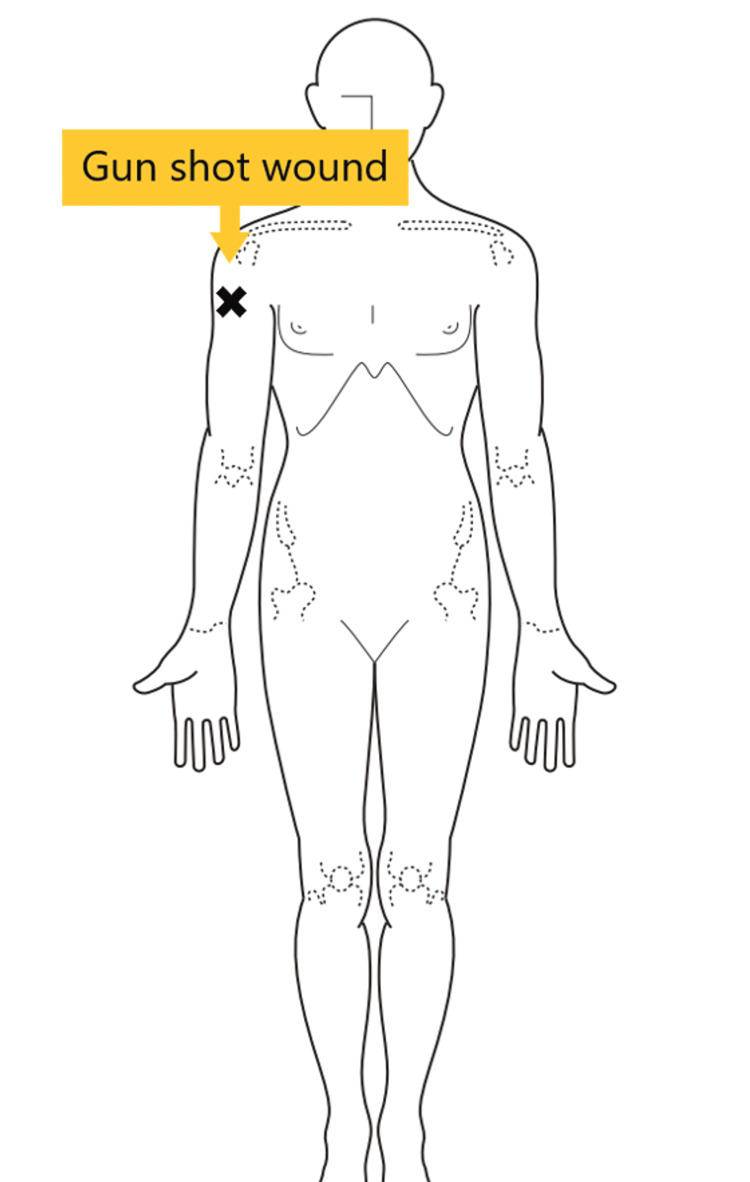
Location of the gunshot wound A single gunshot wound was identified in the right upper extremity.

The patient was alert with stable hemodynamics (blood pressure 120/80 mmHg, heart rate 78 bpm, Glasgow coma scale 15). A single gunshot wound was noted on the right upper arm's outer side (Figure 1). There was no limb paralysis or active bleeding. A 26 Fr chest drain was placed for the right pneumothorax.

Laboratory Findings

The patient's laboratory results indicated mild anemia with a hemoglobin level of 11.2 g/dL, a normal white blood cell count of 11.2 x 10^3/uL, and a platelet count of 219 x 10^3/uL. Creatinine was 0.93 mg/dL. Blood urea nitrogen was normal at 10 mg/dL. Coagulation tests showed a prothrombin time international normalized ratio of 1.0 and an activated partial thromboplastin time of 23.7 seconds. Lactic acid was 4.2 mmol/L.

Imaging Findings

**Figure 2:**
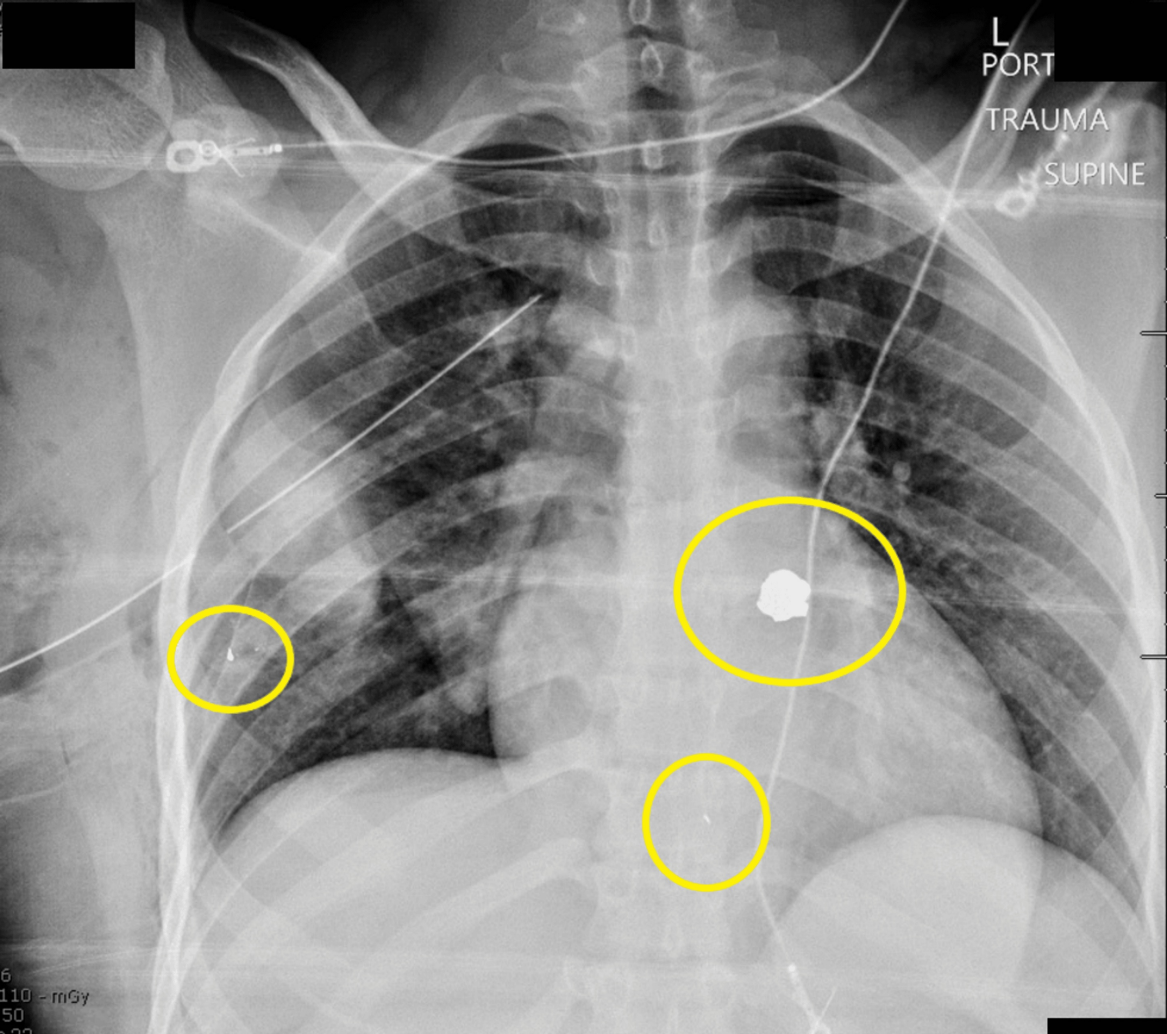
Preoperative CXR Preoperative CXR reveals three bullet fragments.

Chest X-ray (CXR): small ballistic fragments in the right chest wall, bullets, and fragments visible within the mediastinum (Figure 2).

**Figure 3:**
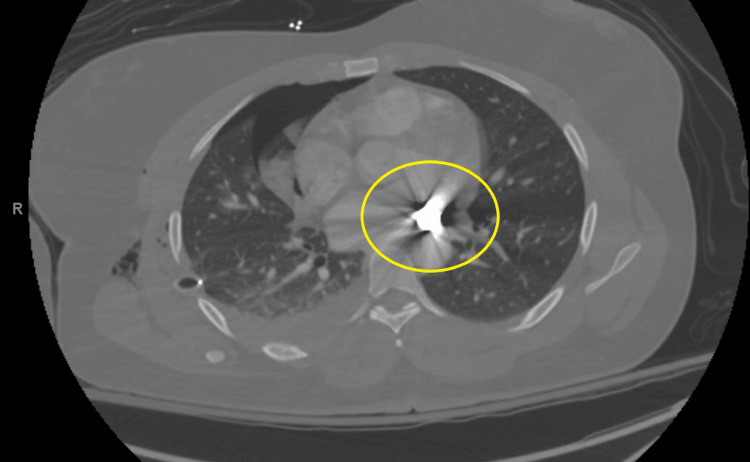
Preoperative CTA A hyperdense object was identified adjacent to the left inferior pulmonary vein.

**Figure 4:**
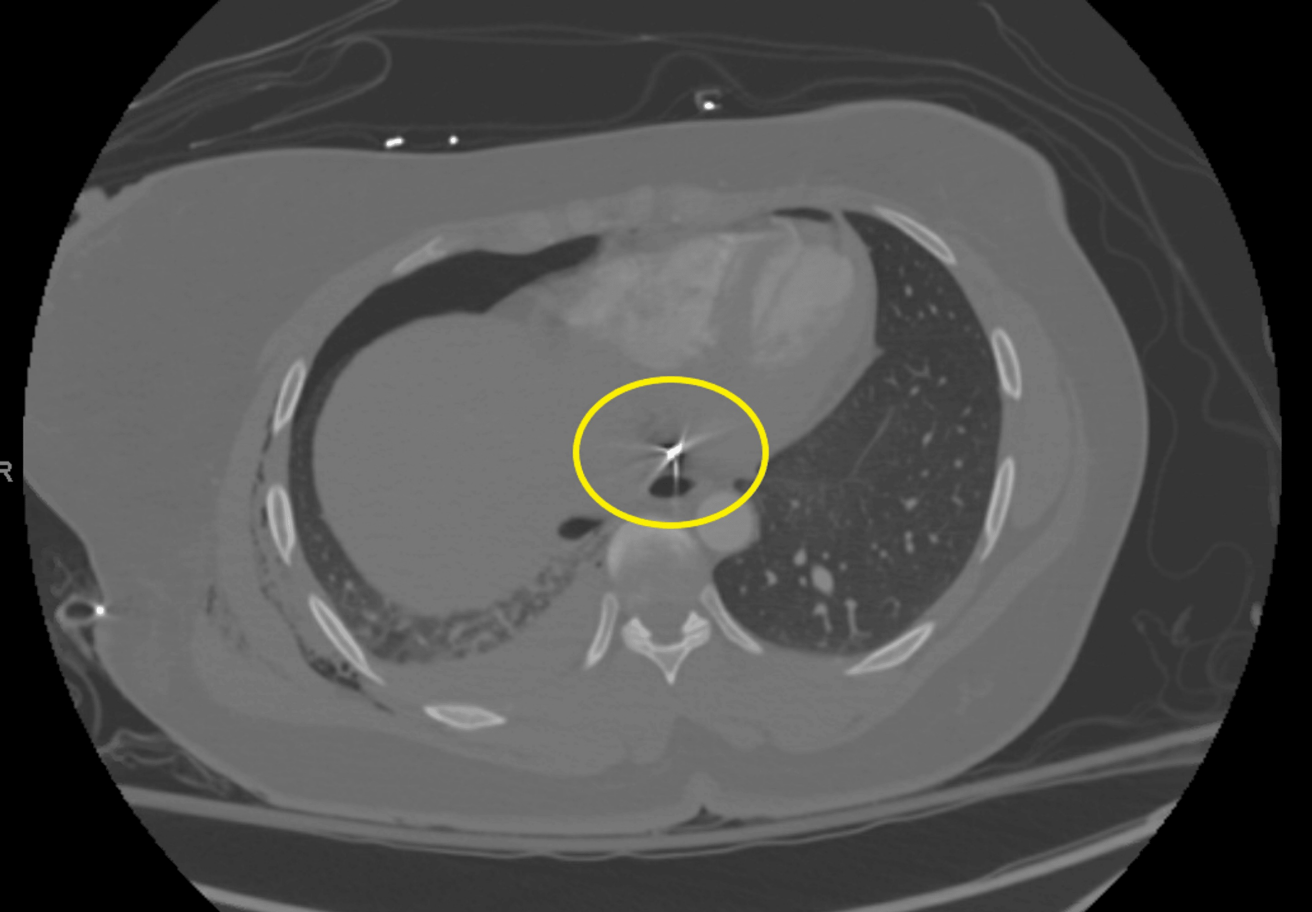
Preoperative CTA An additional hyperdense object was located in proximity to the inferior vena cava.

CTA: hyperdense object near the left inferior pulmonary vein, likely the bullet, and another hyperdense object near the inferior vena cava, presumed to be a fragment. A small bullet fragment is also seen in the right chest wall. Minimal pericardial fluid was noted (Figures 3, 4).

Transthoracic echocardiography (TTE)/transesophageal echocardiography (TEE): normal size and function of the left and right ventricles, left and right atria. No pericardial effusions or intracardiac bullets were detected.

Intraoperative Findings

**Figure 5:**
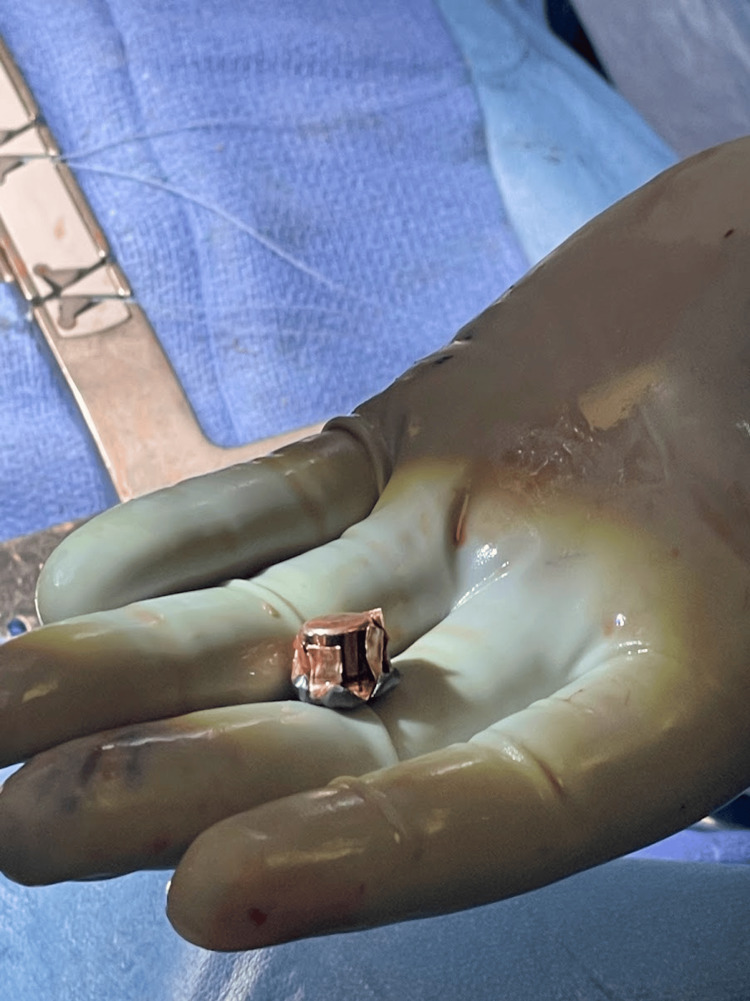
Extracted bullet fragment Two bullet fragments were extracted from the pericardium.

The patient underwent a median full sternotomy. A hole in the right pleural cavity, leading to a pericardial defect, was noted. Upon opening the pericardium, a small amount of bright red pericardial effusion was observed. A 4 cm transverse laceration in the right ventricle at the pericardium defect site and a 1 cm laceration near the left anterior descending artery were found. No active bleeding was noted. These injuries appeared to be caused by the bullet (Video 1). A bullet and its fragment were found behind the heart (Figure 5). The right ventricular lacerations were covered with hemostatic agents. No other injuries were detected. 24 Fr Blake Drains tubes were placed in the mediastinal cavity. The pericardium was closed with a Gore-Tex membrane, and the chest was closed with sternal wires and plates.

**Video 1:** Intraoperative findings A 4 cm transverse laceration in the right ventricle at the pericardium defect site was found.

**Figure 6:**
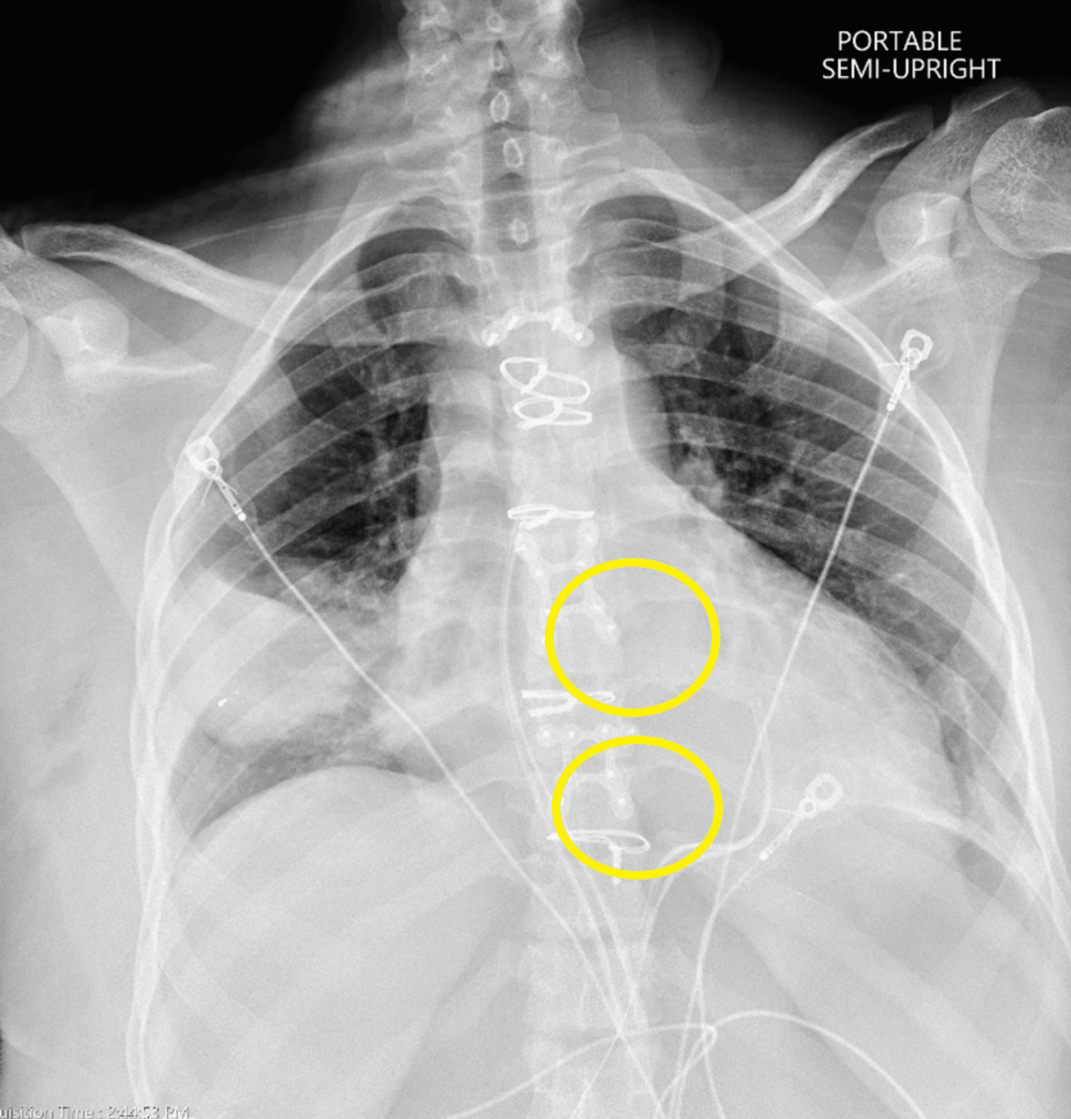
Postoperative CXR The postoperative CXR demonstrates the removal of two bullet fragments.

The patient's hemodynamics were stable postoperatively and progressed favorably. The patient self-extubated 12 hours after the surgery and is currently undergoing rehabilitation. The postoperative CXR revealed the successful removal of two bullet fragments, with one fragment remaining embedded in the chest wall (Figure 6). The postoperative TTE demonstrated preserved function of both the left and right ventricles, as well as normal valve motion.

## Discussion

In previous reports, there have been cases where individuals survived gunshot wounds to the heart, leading to pericardial effusion [3,4]. However, there were no reported cases of a bullet grazing the heart without causing pericardial effusion and while maintaining stable hemodynamics. This case report contributes to the knowledge of gunshot wound treatment by presenting clear preoperative and intraoperative findings.

Preoperative TTE and TEE revealed that there was no bullet inside the heart chambers or in the pericardium. Therefore, even if a bullet is not detected in the pericardium with TTE or TEE, its presence should not be ruled out. In this case, there was almost no pericardial effusion, although it is common to assume pericardial effusion when a bullet is present in the pericardium. This suggests that the possibility of a bullet being in the pericardial cavity should not be dismissed, even when there is no significant pericardial effusion.

The gunshot wound was only on the outer right upper arm, suggesting that the bullet entered the body through the right upper arm, passed through the right thoracic cavity, penetrated the right pleura and pericardium, grazed the anterior surface of the right ventricle, and then decelerated and remained in the pericardium. It is particularly interesting that the bullet, having passed through the pericardium with enough speed to penetrate it, did not pierce the opposite pericardium and remained in the pericardium. There was no apparent damage to the opposite pericardium. It was also extremely fortunate that the bullet did not damage the anterior descending coronary artery and the right coronary artery, nor did it penetrate the opposite pericardium and damage the descending aorta.

In this case, the bullet in the pericardium was removed, but there are past reports where bullets were not removed and were instead monitored over time. Kaya et al. reported a case where a patient diagnosed with a gunshot wound only to the left arm developed cardiac tamponade, leading to the discovery of a bullet in the pericardium, which was then treated conservatively [3]. Another report described conservative treatment of a bullet that penetrated the superior vena cava and entered the myocardium of the right ventricle [4].

## Conclusions

This case report presents a rare instance of survival following a cardiac gunshot wound, emphasizing the need for careful assessment and surgical intervention even when clinical signs like significant pericardial effusion are absent. The successful management of this case, despite the unusual absence of common clinical indicators, underlines the importance of not dismissing the possibility of bullet retention in the pericardium based solely on imaging findings. This contributes valuable insights to the treatment strategies for traumatic cardiac injuries, demonstrating that positive outcomes are achievable even in complex gunshot wound scenarios.
