# Correction: Survival After Cardiac Laceration From a Gunshot Wound: A Rare Case Report

**DOI:** 10.7759/cureus.c213

**Published:** 2025-02-27

**Authors:** Yusuke Tsukioka, Atsushi Nemoto, Raquel Salazar

**Affiliations:** 1 Cardiothoracic Surgery, University of Chicago Medicine, Chicago, USA; 2 Cardiac Support, University of Chicago Medicine, Chicago, USA

The mortality rate (24.5%) was mislabeled as the gunshot wound survival rate in the abstract and introduction sections. This has been corrected.

